# Predictors of condom use behavior among men who have sex with men in China using a modified information-motivation-behavioral skills (IMB) model

**DOI:** 10.1186/s12889-019-6593-8

**Published:** 2019-03-04

**Authors:** Hongbo Jiang, Xiaobin Chen, Jing Li, Zhimin Tan, Weibin Cheng, Yi Yang

**Affiliations:** 1 Department of Epidemiology and Biostatistics, School of Public Health, Guangdong Pharmaceutical University, No. 283 Jianghai Road, Haizhu District, Guangzhou, China; 2 Department of AIDS/STD Control and Prevention, Guangzhou Center for Disease Control and Prevention, No. 1 Qide Road, Baiyun District, Guangzhou, China

**Keywords:** Men who have sex with men, Condom use, HIV, Information-motivation-behavioral skills model, Multilevel factors

## Abstract

**Background:**

Men who have sex with men (MSM) are at high risk for human immunodeficiency virus (HIV) infection in China. Correct and consistent condom use is one of the most effective strategies for preventing the spread of HIV. This study developed a modified Information-Motivation-Behavioral Skills (IMB) model to predict condom use behavior among Chinese MSM.

**Methods:**

A cross-sectional study was conducted to collect data using self-administered electronic questionnaire. Participants were recruited from HIV Voluntary Counseling and Testing clinics in six district Centers for Disease Control and Prevention in Guangzhou and two community-based HIV service centers (Lingnan Partners and Zhitong Charity) from May to September 2017. Structural equation modeling was performed to develop the modified IMB model with extended multilevel factors.

**Results:**

Among the 976 MSM included, 52.05% had engaged in anal intercourse with a condom every time. The final modified IMB model fitted the data more ideally than the conventional model. The final modified IMB model revealed that behavioral skills positively contributed directly to condom use (*β* = 0.385, *p* < 0.001) and partially mediated the associations between information (*β* = 0.106, *p* = 0.005) and motivation (*β* = 0.390, *p* < 0.001) and condom use. Regarding the extended multilevel factors, education, income, receiving HIV prevention services, sexual partner seeking behavior, depression, intimate partner violence, and child sexual abuse had indirect impacts on condom use that were mediated by information, motivation, and/or behavioral skills (*p* < 0.05). All paths from the latent variable to the corresponding observed variables were statistically significant (*p* < 0.001).

**Conclusion:**

The modified IMB model with extended multilevel factors could serve as a theoretical framework for behavioral interventions for condom use among Chinese MSM. Further prospective studies are needed to examine the predictive power of the modified IMB model.

**Electronic supplementary material:**

The online version of this article (10.1186/s12889-019-6593-8) contains supplementary material, which is available to authorized users.

## Background

Antiretroviral therapy (ART) has greatly reduced HIV-related mortality since 1996. While human immunodeficiency virus (HIV) infection/acquired immune deficiency syndrome (AIDS) remains a leading cause of death and a disease burden in many countries and regions [1]. Men who have sex with men (MSM) continue to have disproportionately high burdens of HIV infection in countries of low-, middle-, and high-income [2]. Notification of HIV cases among MSM in China rose from 2.5% in 2006 to 25.8% in 2014 [3]. The results of a multisite cross-sectional study reported high HIV prevalence of 9.9% among MSM in China [4]. Unprotected anal intercourse (UAI) is a common high-risk behavior and reason for HIV infection among MSM [5]. Evidence from a recent meta-analysis showed that the proportion of UAI with any male partner among MSM in China was 53% (95% *confidence interval [CI]*: 51–56%) [6]. One serial cross-sectional study presented that the proportion of UAI was 58.4% during the last 6 months among MSM in Guangzhou [7]. Correct and consistent condom use, is one of the most effective strategies for preventing the spread of HIV among general and high-risk populations [8]. Strengthening this behavior among MSM in China is thus an important issue for research.

Evidence shows that new public health and/or health-promotion interventions based on social and behavioral science theories are more effective than those lacking a theoretical framework [9], this is because these interventions are tailored towards addressing the identified predictors of the health behavior of interest. Specifically, the Information-Motivation-Behavioral Skills (IMB) model has been found to significantly predict condom use among MSM [10], female sex workers [11], people attending sexually transmitted infection (STI) clinics [12], and students [13]. Consequently, interventions designed along IMB constructs have been found to increase condom use among MSM [14], HIV-infected patients [15], and students [16]. The IMB model proposes that information, motivation, and behavioral skills are fundamental determinants of HIV-preventive behaviors, e.g. condom use [17]. According to the model, possession of adequate information coupled with a strong motivation to act on the information propels the desired behavioral skills, which in turn initiate and sustain condom use. The behavioral skills that directly influence condom use also partially mediate the associations between information/motivation and condom use [17–19].

Although the components of the IMB model influence one another, studying them in isolation from other social influences of behavior yields less predictive power [20]. The IMB model in its non-extended form relies only on psychological or individual-level influences, whereas social factors have a significant influence on behaviors [20, 21]. Meta-analyses of behavioral intervention show that individual-level theories cannot explain the heterogeneity among study outcomes [22]. Thus, researchers have suggested extending the model by incorporating other social factors that influence preventive behaviors [20, 21]. Modified IMB models with extended constructs have been found to explain more variance in HIV-preventive behavior than the original non-extended model [20, 21, 23, 24]. However, existing literature has insufficiently integrated these social factors and explored their interactions [25]. Recent efforts have been made to expand from individual-level factors to multilevel factors at different ecological levels. The multiple domain model (MDM) suggests that factors outside of the individual are modeled as factors shaping one’s behavior; these factors including structural factors, personality, and social environment situational/contextual variables [26]. The network-individual-resource model (NIRM) proposes that individual behavior changes for HIV prevention can be supported and sustained by the tangible and mental resources possessed by individuals and networks and that the outcomes may be resilient over time and disseminated more broadly [27]. The dynamic social systems model (DSSM) focuses on the structures that people face through interactions at the micro-, meso-, and macro-levels [28]. The Transmission Reduction Intervention Project (TRIP) suggests that HIV prevention can be greatly improved by using social science as an integrative tool in transdisciplinary research and practice [29]. Another review recommended a multilevel approach to HIV-related behavior changes, which suggested that the HIV risk and AIDS care involve complex behaviors beyond individual-level factors that are influenced by multilevel factors [30]. These multilevel factors include individual-level factors, interpersonal/network-level factors, and structural-level factors. Among the individual-level factors, income, education and depression were found to be associated with condom use [23, 31, 32], and these factors were integrated into a modified IMB model [20, 23]. The interpersonal/network-level factors such as child sexual abuse (CSA) and intimate partner violence (IPV) tended to co-occur and increased the risk of UAI together with depression [32–34]. With use of Internet and mobile applications, an increasing number of MSM seek sexual partners online. Sexual partner seeking behavior, which is another interpersonal/network-level factor, may also impact condom use behavior [35]. Moreover, structural-level factors, such as access to HIV prevention and care services, may also have a positive effect on condom use [36]. However, the original IMB model has no role for interpersonal/network-level or structural-level factors. Thus, an extension of the IMB model with interpersonal/network-level, structural-level, and more individual-level factors may be meaningful for improvement of the predictive power for the condom use behavior.

However, less is known about the application of modified IMB models for predicting condom use among MSM in China. Based on the high proportion of UAI [6, 7], increasing HIV prevalence [3, 4], and lack of evidence for the influence of the modified IMB model among MSM in China, we conducted this cross-sectional study to integrate the multilevel factors into the conventional IMB model and built a modified IMB model for predicting the condom use among MSM in China. The aim of this study is to provide a theoretical framework for safe sex behavioral intervention.

## Methods

### Participants

MSM were recruited from the HIV Voluntary Counseling and Testing (VCT) clinics of six district Centers for Disease Control and Prevention (CDCs) in Guangzhou (Baiyun, Conghua, Panyu, Huadu, Huangpu, and Zengcheng District), and two community-based HIV service centers (Lingnan Partners in Tianhe and Zhitong Charity in Yuexiu District) from May to September 2017 using a convenience sampling method. The inclusion criteria were as follows: (1) male aged 18 years and above, (2) had anal/oral intercourse with a man within the past 12 months, and (3) lived in Guangzhou for more than 3 months. Individuals who had obvious psychological or mental illnesses, dysnoesia, a foreign nationality, or refused to participate in the study were excluded. Individuals with debilitating illnesses and hearing or visual impairment were also excluded. The sample size was calculated using the formula [N=${\mathrm{Z}}_{\upalpha}^2\mathrm{P}\left(1-\mathrm{P}\right)/{\mathrm{d}}^2\times deff$]. With a proportion of consistent condom use among MSM in Guangzhou (*P*) of 41.6% [7], precision error (*d*) of 0.1 *P*, 95% confidence level, and design effect (*deff*) of 1.5 due to non-probability sampling [37], the minimum sample size required for this study was calculated to be 890 after taking a non-response rate of 10% into consideration. MSM who met the inclusion criteria filled out the electronic questionnaire (Additional file 1). After completing the questionnaire, the participants underwent HIV consultations and testing. They also received a gift (condoms and personal lubricants) as compensation for their time. A total of 1174 MSM completed the survey during the study period, but the analysis was based on 976 who reported having anal intercourse within the past 6 months.

### Measures

#### Demographics and sexual characteristics

Information was collected on age, birthplace, duration in Guangzhou, marital status, education, monthly income, occupation, sexual orientation, sexual role, and sexual partner seeking behaviors.

#### HIV prevention services

Three questions were used to assess whether the participants had received HIV prevention services within the last year. Such services include (1) condom promotion and distribution or HIV voluntary counseling and testing, (2) community-based methadone maintenance treatment or cleaning needle provided or exchanged, and (3) peer education for HIV prevention. Responses with “Yes” to any of these questions indicated receiving the HIV prevention services.

#### Depression

Depression was assessed using the Zung self-rating depression scale (SDS) [38]. The SDS index is derived by dividing the sum of the raw scores obtained on the 20 items by the maximum possible score of 80 and is expressed as a decimal point. An SDS index of 0.5 or above indicates depression (Cronbach’s α = 0.80).

#### CSA

CSA was assessed with the following four questions: (1) “Did you have any sexual experiences (someone exposed his genitals or masturbated in front of you or attempted to have or had oral or anal sex with you) before 12 years of age?” (2) “Was the person you had the sexual experience with an adult or someone at least 5 years older than you?”; (3) “Between 12 and 16 years of age, did you have any unwanted sexual experiences?”; and (4) “Between 12 and 16 years of age, did you have any sexual experiences (wanted or unwanted) with an adult or someone who was at least 5 years older than you?” [33]. Responses with “Yes” to any of these questions indicated a CSA experience.

#### IPV

IPV was measured as ever experiencing an intimate partner who (1) “threatened to stop helping you with money or housing”; (2) “damaged or destroyed your property”; (3) “threatened to tell others about your sexual orientation”; (4) “verbally threatened to physically harm someone you cared for”; (5) “verbally threatened to harm you physically or emotionally”; (6) “hit you or threw something at you”; or (7) “forced you to have unwanted sex” [34]. Participants who responded with “Yes” to any of these questions were defined as a victim of IPV.

#### Information

Eight questions about general awareness of HIV/AIDS were used to assess prevention behavior (condom use) information among Chinese MSM under the guideline of the national AIDS sentinel surveillance program: (1) “Is AIDS an incurable serious infectious disease?”; (2) “Are MSM the most serious victims of AIDS in China?”; (3) “May a person who looks healthy carry HIV?”; (4) “Will infection with other sexually transmitted diseases increase the risk of HIV infection?”; (5) “Can correct condom use reduce the risk of HIV infection and transmission?”; (6) “Will the use of new drugs (methamphetamine, ecstasy, ketamine, etc.) increase the risk of HIV infection?”; (7) “Should HIV consultation and testing be actively sought after high-risk behaviors (needle-sharing drug abuse/unsafe sex, etc.)?”; (8) “Does deliberately spreading AIDS bear legal liability?”. These questions were correctly answered as “Yes”, but wrongly answered or unknown as “No” (Cronbach’s α = 0.57). Individuals answering six or more questions correctly were defined as having adequate HIV-related knowledge [39].

### Motivation

The motivation construct was measured using questions that assessed personal attitudes towards condom use, subjective norms and behavioral intentions regarding condom use [17, 18]. Personal attitudes towards condom use were assessed by four items (e.g., “Discussion with my sexual partner about safe sex before sexual intercourse during the next month would be...”) adapted from published scales using a 5-point Likert scale ranging from 1 (negative evaluation) to 5 (positive evaluation). A composite score was obtained by summing the responses to the four items, with higher scores indicating more positive personal attitudes towards condom use (Cronbach’s α = 0.96).

Subjective norms regarding condom use were assessed by four items (e.g., “Most people who are important to me think I should discuss safe sex with a sexual partner before sexual intercourse during the next month”) using a 5-point Likert scale ranging from 1 (negative evaluation) to 5 (positive evaluation). A composite score was obtained by summing the responses to the four items, with higher scores indicating more positive subjective norms regarding condom use (Cronbach’s α = 0.96).

Behavioral intentions regarding condom use were assessed by four items (e.g., “If I have sex during the next month, I intend to discuss safe sex with a sexual partner before sexual intercourse”) using a 5-point Likert scale ranging from 1 (negative evaluation) to 5 (positive evaluation). A composite score was obtained by summing the responses to the four items, with higher scores indicating more positive behavioral intentions regarding condom use (Cronbach’s α = 0.96).

Personal attitudes towards condom use, subjective norms and behavioral intentions regarding condom use comprised the latent construct of the motivation to adopt HIV-preventive behaviors (Cronbach’s α = 0.96).

### Behavioral skills

The behavioral skills comprised the perceived difficulty and perceived effectiveness of HIV-preventive behaviors [17, 18].

The perceived difficulty of AIDS preventive behaviors was assessed by six items (e.g., “How hard would it be for you to buy condoms”) using a 5-point Likert scale ranging from 1 (very hard) to 5 (very easy). A composite score was obtained by summing the responses to the six items, with higher scores indicating easier adoption of HIV-preventive behaviors (Cronbach’s α = 0.78).

The perceived effectiveness of AIDS preventive behaviors was assessed by five items (e.g., “How effectively could you discuss safe sex with a sexual partner before sexual intercourse”) using a 5-point Likert scale ranging from 1 (very ineffectively) to 5 (very effectively). A composite score was obtained by summing the responses to the five items, with higher scores indicating more effective adoption of HIV-preventive behaviors (Cronbach’s α = 0.95).

Perceived difficulty and perceived effectiveness comprised the latent construct of the behavioral skills associated with HIV-preventive behaviors (Cronbach’s α = 0.73).

### Condom use

The participants were asked how often they had used condoms during anal sex over the past 6 months using a 5-point Likert scale ranging from 1 (never) to 5 (always). A higher score indicated stronger commitment to HIV-preventive behaviors. Response with 5 (always) was defined as consistent condom use.

### Statistical analysis

The data were abstracted from the electronic questionnaire platform. After data checking, frequencies and percentage were used to present categorical characteristics, and means (M) and standard deviations (SDs) were used for continuous characteristics of the participants. Pearson’s correlation analysis was conducted to examine the correlations between the components of the modified IMB model with extended associated factors [20]. Structural equation modeling was performed to construct conventional and modified IMB models using a weighted least squares (WLS) estimator [40]. Model fit was assessed using the *χ*^*2*^ and degree of freedom ratio (χ^2^/*df* ratio), root mean square error of approximation (RMSEA), goodness of fit index (GFI), and adjusted goodness of fit index (AGFI) [41–43]. A good fit was indicated when the *χ*^2^/*df* ratio ≤ 3 and RMSEA ≤0.05 [41]. The GFI and AGFI were used to explain variance and were considered acceptable when the values were ≥ 0.90 [42]. The data analyses were performed using SAS Version 9.4 (SAS Institute Inc., Cary, NC, USA). All hypothesis tests were 2-tailed with α = 0.05.

## Results

### Social, sexual and psychological characteristics of the participants

In total, 976 MSM who had anal intercourse during the past 6 months were included in the current study. The mean age was 28.35 ± 6.83 years with a range from 18 to 67 years. Approximately half (50.61%) of the participants were recruited from Lingnan Partners, 31.35% from Zhitong Charity, and 18.03% from the six selected VCT clinics. The majority of the MSM participants had lived in Guangzhou for more than 12 months (88.52%) and were college educated or above (75.31%), unmarried (86.99%), and employed (79.61%). Approximately one-third of the participants were residents of Guangzhou (35.66%), followed by residents of other cities in Guangdong Province (33.50%), and of other provinces (30.84%). Approximately half (50.10%) of the participants earned more than 5000 Yuan (equivalent to U.S. $725) per month.

Among the 976 participants, 74.08% identified themselves homosexual. Regarding the anal sex role, 157 (16.09%) reported themselves as undertaking the insertive anal sex role with their male partners, 158 (16.19%) reported themselves as undertaking the receptive anal sex role, and the rest (67.73%) were versatile. Most (89.96%) of the participants sought homosexual partners on the Internet. Approximately two-thirds (63.83%) of the participants received HIV prevention services within the last year. The proportions of participants who reported consistent condom use, CSA, IPV, and depression were 52.05, 26.95, 13.22, and 43.24%, respectively (Table 1).

**Table 1:** Sociodemographic, sexual and psychosocial characteristics of 976 men who have sex with men in Guangzhou, China

Characteristics	N(%^a^)	Characteristic	N(%^a^)
Age (years)		Sexual orientation	
18~	242(24.80)	Homosexual	723(74.08)
24~	287(29.40)	Heterosexual	4(0.41)
28~	205(21.00)	Bisexual	208(21.31)
32~67	242(24.80)	Unsure	41(4.2)
Birthplace		Sexual role	
Guangzhou City	348(35.66)	Insertive only	157(16.08)
Other cities in Guangdong province	327(33.50)	Receptive only	158(16.19)
Other provinces	301(30.84)	Versatile	661(67.73)
Duration in Guangzhou(months)		Seeking sexual partners ^c^	
<12	112(11.48)	Internet	878(89.96)
≥12	864(88.52)	Non-Internet	98(10.04)
Education		Condom use during anal sex	
≤Middle school	66(6.76)	Never	38(3.89)
High school or equivalent	175(17.93)	Occasionally	114(11.68)
≥College	735(75.31)	Sometimes	75(7.69)
Marital status		Often	241(24.69)
Unmarried	849(86.99)	Always	508(52.05)
Married/cohabitation	104(10.66)	HIV prevention service	
Divorced/widowed	23(2.35)	Yes	623(63.83)
Monthly income (Yuan)		No	353(36.17)
0	118(12.09)	Child sexual abuse	
1~	96(9.84)	Yes	263(26.95)
3001~	273(27.97)	No	713(73.05)
5001~	320(32.79)	Intimate partner violence	
>10,000	169(17.31)	Yes	129(13.22)
Occupation ^b^		No	847(86.78)
Student	146(14.96)	Depression	
Employed	777(79.61)	Yes	422(43.24)
Other	52(5.33)	No	554(56.76)
Total	976(100.00)	Total	976(100.00)

^a^Because of rounding, not all percentages total 100

^b^One case was missing occupation information. Other refers to part-time, unemployed, retired, and so on

^c^Internet refers to websites or smart phone applications. Non-Internet refers to real places such as a bar, dance hall, public baths, and so on

### Information, motivation, and behavioral skills

The mean HIV knowledge score was 6.59 ± 1.35. For motivation, the mean condom attitudes, subjective norms and behavioral intentions scores were 17.12 ± 3.83, 17.49 ± 4.33 and 18.00 ± 3.40, respectively. Regarding behavioral skills, the mean scores for perceived difficulty and effectiveness were 25.87 ± 3.80 and 19.76 ± 5.67, respectively. The frequency of condom use when having anal sex during the last 6 months was 4.09 ± 1.19 (Table 2).

**Table 2:** Mean scores of constructs of the IMB model among 976 men who have sex with men in Guangzhou, China

Construct	M ^a^	SD ^b^
Information		
Knowledge (0–8)	6.59	1.35
Motivation		
Personal attitudes (4–20)	17.49	4.33
Subjective norms (4–20)	17.12	3.83
Behavioral intentions (4–20)	18.00	3.40
Behavioral skills		
Perceived difficulty (6–30)	25.87	3.80
Perceived effectiveness (5–25)	19.76	5.67
HIV preventive behavior (Condom use: 1–5)	4.09	1.19

^a^*M* mean

^b^*SD* standard deviation

### Correlations among constructs in the IMB model

Condom use was positively associated with education, income, seeking sexual partners online, receiving HIV prevention services, personal attitudes, subjective norms, behavioral intentions, perceived difficulty, and perceived effectiveness (*p* < 0.01), and was negatively associated with depression and IPV (*p* < 0.01) (Table 3).

**Table 3:** Pearson’s correlation between constructs of the modified IMB model among 976 men who have sex with men in Guangzhou, China

No.	Construct	1	2	3	4	5	6	7	8	9	10	11	12	13	14
1	Education	1													
2	Income	0.24^***^	1												
3	Sexual partner seeking behavior	0.14^***^	0.06	1											
4	Depression	−0.14^***^	− 0.13^***^	− 0.06	1										
5	Intimate partner violence	− 0.05	− 0.03	− 0.12^***^	0.09^**^	1									
6	Child sexual abuse	−0.10^**^	−0.09^**^	−0.06	0.13^***^	0.13^***^	1								
7	HIV prevention services	0.10^**^	0.01	0.02	−0.09^**^	0.02	0.01	1							
8	Information	0.09^**^	0.07^*^	0.04	−0.06^*^	0.04	−0.01	0.09^**^	1						
9	Personal attitudes	0.20^***^	0.12^***^	0.07^*^	−0.19^***^	−0.04	−0.08^*^	0.01	0.12^***^	1					
10	Subjective norms	0.09^**^	0.02	−0.03	−0.13^***^	−0.05	− 0.03	0.02	0.04	0.57^***^	1				
11	Behavioral intentions	0.16^***^	0.10^**^	0.02	−0.13^***^	0.01	−0.05	0.01	0.10^***^	0.64^***^	0.65^***^	1			
12	Perceived difficulty	0.19^***^	0.19^***^	0.06	−0.23^***^	−0.11^***^	−0.05	0.13^***^	0.09^**^	0.41^***^	0.41^***^	0.44^***^	1		
13	Perceived effectiveness	0.12^**^	0.14^**^	0.16^***^	−0.32^***^	−0.07^*^	−0.15^***^	0.09^**^	0.11^**^	0.24^***^	0.18^***^	0.25^***^	0.37 ^***^	1	
14	Condom use	0.17^***^	0.13^***^	0.10^**^	−0.12^***^	−0.09^**^	−0.05	0.09^**^	0.05	0.18^***^	0.15^***^	0.14^***^	0.29^***^	0.32^***^	1

### Model estimation

#### Conventional IMB model

**Fig. 1:**
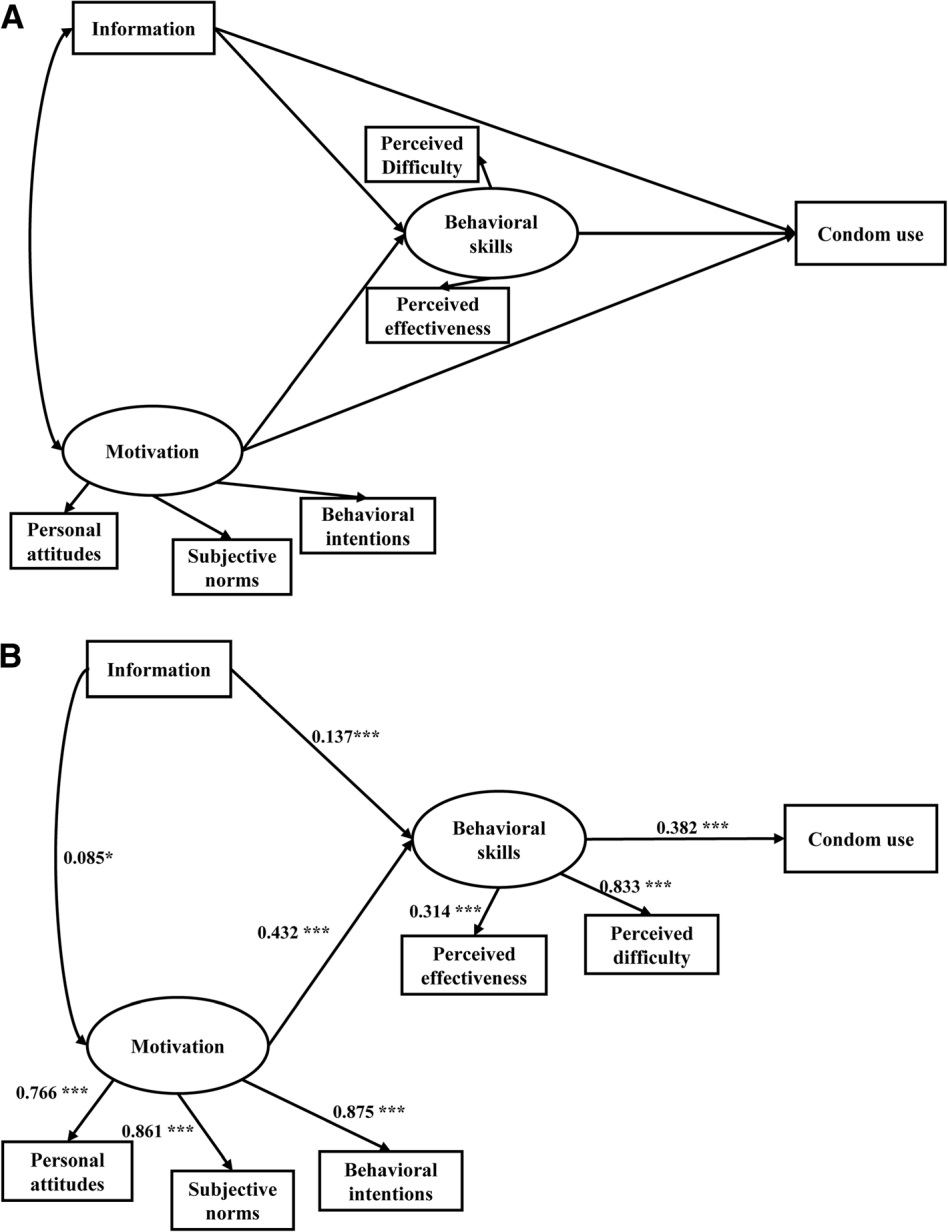
The initial and final conventional IMB models with standardized path coefficients among 976 men who have sex with men in Guangzhou, China. **a**: The initial conventional IMB model. **b**: The final conventional IMB model. *P < 0.05, ** *P* < 0.01, *** *P* < 0.001

In the final conventional IMB model, behavioral skills positively and directly predicted safe sex behavior (standardized path coefficient *β* = 0.382, *p* < 0.001) and partially mediated the associations between information (*β* = 0.137, *p* < 0.001) and motivation (*β* = 0.432, *p* < 0.001) and condom use (Fig. 1). All paths from the latent variable to the corresponding observed variables were statistically significant (*p* < 0.001). However, the final conventional IMB model did not fit the data ideally, with a *χ*^*2*^/*df* of 3.201, which was not within the acceptable rang of 3 or less, although the RMSEA was 0.048 < 0.05, GFI was 0.976 > 0.90, and AGFI was 0.945 > 0.90 (Table 4). The total effect of all IMB constructs on condom use was 0.599.

**Table 4:** The model fit of the conventional and modified IMB models among 976 men who have sex with men in Guangzhou, China

Index	Criterion	Conventional IMB	Modified IMB
*χ*^*2*^/*df* ^a^	< 5.00	3.201	2.801
RMSEA	< 0.05	0.048	0.043
GFI	> 0.90	0.976	0.98
AGFI	> 0.90	0.945	0.96

^a^*χ*^*2*^/*df*: *χ*^*2*^ and degree of freedom ratio; RMSEA: root mean square error of approximation; GFI: goodness of fit index; AGFI: adjusted goodness of fit index

#### Modified IMB model

**Fig. 2:**
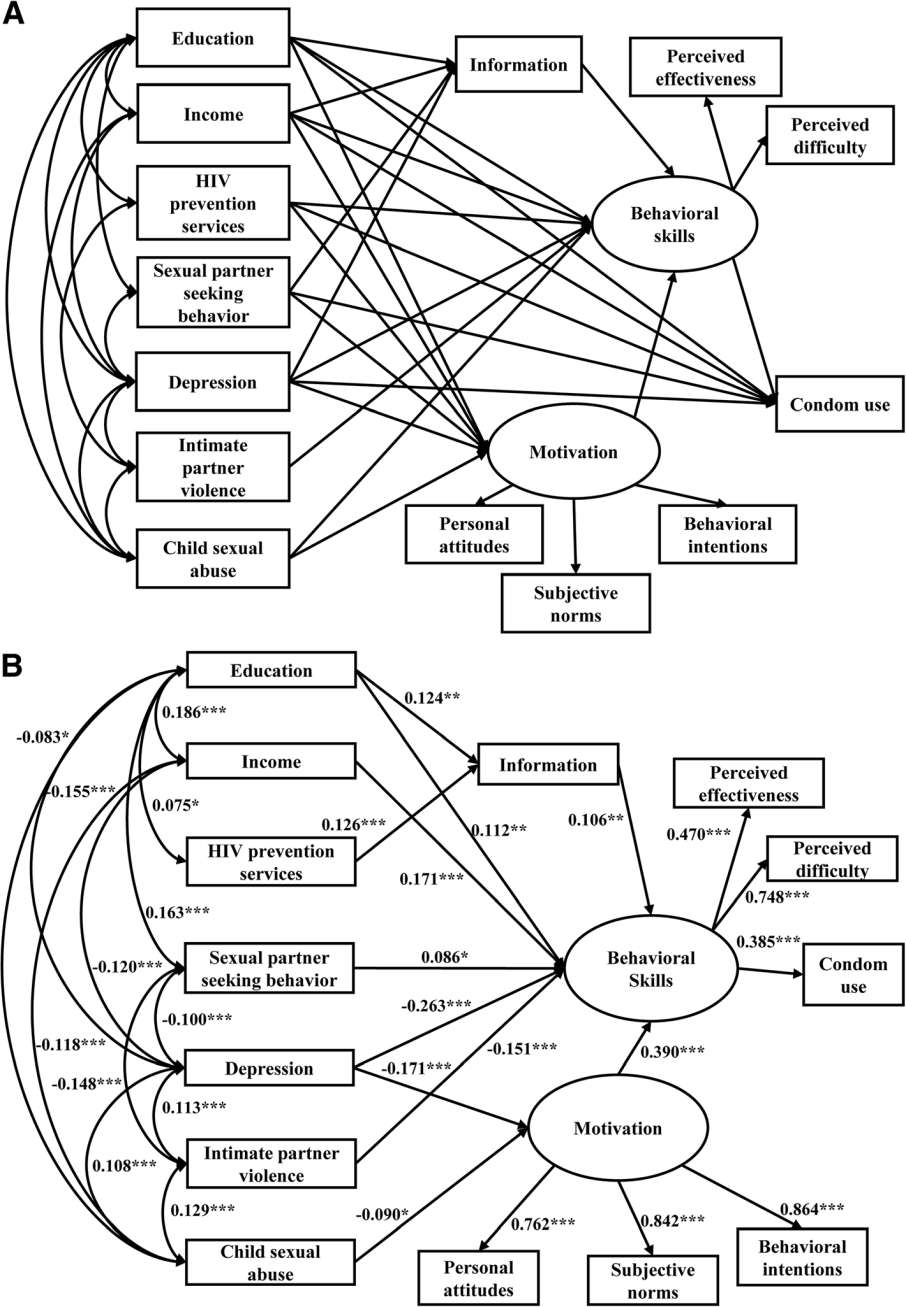
The initial and final modified IMB models with standardized path coefficients among 976 men who have sex with men in Guangzhou, China. **a**: The initial modified IMB model. **b**: The final modified IMB model. *P < 0.05, ** *P* < 0.01, *** *P* < 0.001

Similar to the final conventional IMB model, the final modified IMB model revealed that behavioral skills positively contributed to safe sex behavior directly (*β* = 0.385, *p* < 0.001) and partially mediated the associations between information (*β* = 0.106, *p* = 0.005) and motivation (*β* = 0.390, *p* < 0.001) and condom use (Fig. 2). Regarding the extended associated factors, higher education (*β* = 0.124, *p* = 0.001) and receiving HIV prevention services (*β* = 0.126, *p* < 0.001) had a positive effect on information. Depression (*β* = − 0.171, *p* < 0.001)) and CSA (*β* = − 0.090, *p* = 0.014) had a negative effect on motivation. Participants who were more educated (*β* = 0.112, *p* = 0.003), had a higher income (*β* = 0.171, *p* < 0.001), and sought sexual partners online (*β* = 0.086, *p* = 0.027) were more likely to have better behavioral skills. Participants with depression (*β* = − 0.263, *p* < 0.001) and a history of IPV (*β* = − 0.151, *p* < 0.001) were less likely to have better behavioral skills. All paths from the latent variable to the corresponding observed variables were also statistically significant (*p* < 0.001). The final modified IMB model fitted the data ideally, with a *χ*^*2*^/*df* of 2.801, RMSEA of 0.043 < 0.05, GFI of 0.980 > 0.90, and AGFI of 0.960 > 0.90 (Table 4). The total effect of all modified IMB constructs on condom use was 0.927.

## Discussion

The current study applied the conventional IMB model and developed a modified IMB model to predict HIV preventive behavior among Chinese MSM. The modified IMB model fitted the data better than the conventional model. The modified IMB model demonstrated that the multilevel factors, such as education, income, depression, CSA, IPV, sexual partner seeking behavior, and access to HIV prevention services, impacted condom use in addition to information, motivation, and behavioral skills.

Correct and consistent condom use is one of the most effective strategies for preventing the spread of HIV among MSM [8]. The proportion of consistent condom use among MSM was 52.05%, which was slightly higher than that (47%) reported in one previous meta-analysis in China [6]. When compared to the result of a serial cross-sectional study conducted among MSM in Guangzhou from 2008 to 2013 [7], the proportion of consistent condom use among MSM increased from 41.6 to 52.05% in 2017, which revealed that the basic HIV control and prevention measures were making progress [44]. However, condom promotion remains a challenge due to the large MSM population and the increasing HIV prevalence among MSM [44, 45]. Moreover, one previous study presented that condom use showed no significant protection when comparing occasional use to no use among MSM having any anal sex with an HIV-positive male partner [46]. Therefore, it is important to develop an HIV prevention program for consistent condom use under the guidance of health promotion theoretical frameworks. The IMB model is one theory that effectively predicts the condom use [10–13] and guides condom promotion interventions in various populations, including MSM [14–16].

According to the original IMB model proposed by Fisher [17, 18], condom use was affected primarily by information, motivation and behavioral skills. Consistent with the original IBM model, behavioral skills were important component in the current study and had a direct effect on condom use. Information and motivation did not appear to contribute to condom use directly as Fisher proposed but instead indirectly mediated behavioral skills. Nevertheless, some other studies also demonstrated that the effect of information and motivation on condom use was not significant [23, 47].

In the current study, 81.25% of the participants had adequate HIV-related knowledge. Only the questions “Can correct condom use reduce the risk of HIV infection and transmission” and “Should HIV consultation and testing be actively sought after high risk behaviors (needle-sharing, drug abuse, unsafe sex, etc.)” were correctly answered by 98.05 and 96.21% of the participants, respectively, which achieved the target (90%) of having adequate HIV-related knowledge among MSM [48]. Thus, more efforts should be made to improve the gaps in HIV-related knowledge among MSM. Our results added to existing literature showing that information does not always have a direct effect on sexual risk behavior. This lack of effect could be due to a ceiling effect, since the overall level of HIV-related knowledge was high and did not yield any additional explanatory power [23]. Moreover, previous studies suggested that adequate HIV-related knowledge appeared to be an insufficient determinant to predict condom use and change risk behavior [23, 49]. Although information did not have a direct influence on condom use, information indirectly contributed to condom use mediated by behavioral skills, and remained a necessary component of HIV prevention interventions, which was consistent with previous studies [11, 20].

Motivation also indirectly contributed to condom use mediated by behavioral skills. Individuals with a positive personal attitude towards condom use and higher subjective norms and intentions regarding condom use were more likely to show more positive motivation to use condoms [12, 13, 20]. It is easy to understand the relationship between a positive attitude and condom use. The subjective norm represented the perceptions of social referents and motivation to comply, which could affect HIV prevention intentions and behaviors [11, 12, 17, 20]. However, previous studies suggested that motivation had a direct effect on condom use among male street laborers [20] and STI clinic patients [12]. Behavioral skills contributed to condom use directly, which revealed that individuals who displayed better skills in preparation, negotiation and practice were more likely to engage in HIV prevention behavior [20]. The current study placed behavioral skills in the role as a mediating factor for all components of the model, which was consistent with some previous studies [12, 20] but inconsistent with other studies [11, 24].

The conventional IMB model focused only on individual-level factors that influenced condom use, and did not fit the data ideally in the current study. According to some multilevel models, the HIV risk and AIDS care behaviors are influenced by multilevel factors at different ecological levels [26–30]. One comprehensive and practical review recommended that multilevel approaches for HIV-related behavior changes could serve as the basis for extending the conventional IMB model with multilevel factors [30]. Beyond individual-level factors, the modified IMB model integrated interpersonal/network-level and structural-level factors to fit the data ideally. Moreover, the total effect of the modified IMB model (0.927) on condom use was much larger than that of the non-extended IMB model (0.599). Therefore, this modified IMB model with multilevel factors was suggested for use as a theoretical framework to guide the behavior interventions and improve the condom use among MSM in China.

The results of the final modified IMB model showed that more educated individuals had more adequate HIV prevention and transmission information [50], which contributed to condom use [32]. However, another study showed that in one personal resource construct, education did not have an impact on information or behavioral skills [23]. Low-income individuals may be more likely to encounter stressors and/or limit their access to resources to buffer against these stressors [51]. Individuals with socioeconomic disadvantages utilize their limited cognitive coping resources in dealing with these excess stressors, especially lack of social support, leading to engagement in condomless sexual behaviors [31]. One modified IMB model suggested that depressed MSM were more likely to have sexual risk behaviors to mitigate distress, which might further compromise motivation for behavior changes [23]. Another modified IMB model presented that the latent variable “psychosocial factors”, including depression as one of the five constructs, was negatively associated with behavioral skills [20], which was consistent with the current results.

CSA and IPV had negative effects on condom use, with the former mediated by motivation and the latter by behavioral skills. Individuals with a CSA experience might foster problematic coping styles and relational instability, resulting in greater exposure to risk opportunities and their condom use decisions were more likely to be affected by their partner’s reaction [52]. They were more likely to think condoms interfered with sexual pleasure and less likely to think condoms were important [53], which was consistent with the negative association between CSA and motivation in the current study. Previous studies suggested that individuals who had experienced IPV were significantly less likely to report having felt able to negotiate condom use [54, 55], which was consistent with the negative association between IPV and behavioral skills in the current study.

Due to technological advances, MSM have multiple platforms for online sex seeking, such as gay-specific forums, chat rooms, and dating websites [56]. Previous studies presented that condom use self-efficacy, which referred to condom acquisition, proper condom use, and negotiation skills, played an important role in condom use behavior among MSM. Higher rates of condom use self-efficacy were associated with lower rates of risky sexual practices [57–59]. The good behavioral skills among the MSM who sought sexual partners online in the current study might be attributed to high condom use self-efficacy among these participants [53]. However, whether seeking sex partners online or offline increases condom use remains controversial. Some previous studies indicated that partners sought online could increase the risk of UAI [35, 60], whereas other studies believed that no difference in UAI existed between gay app users and non-app users [56, 61]. Nevertheless, the fact that most (89.96%) of the participants sought homosexual partners online and that 46.81% of the MSM reported UAI in the current study suggested that websites and gay apps were both risk environments and that Internet-based behavioral interventions were necessary. HIV prevention services provided adequate HIV prevention and transmission knowledge and thus had a positive effect on information that indirectly resulted in condom use. However, a gap existed between the target of more than 90% coverage of HIV prevention services [48] and the fact that only approximately two-thirds (63.83%) of the MSM received HIV prevention services. Therefore, more efforts should be made to expand the coverage of HIV prevention services among MSM in China.

Despite these strengths, several limitations should be noted when interpreting and generalizing the results. First, causal inference remained ambiguous due to the cross-sectional nature of this study [12]. Thus, prospective studies are called for to further confirm the effects of the modified IMB model components on HIV-preventive behavior. Second, the convenience sampling approach may limit the generalizability [23]. Nevertheless, the six VCT clinics and the two popular community-based HIV service centers, which covered 8 of the 11 districts in Guangzhou, are believed to have provided a relatively adequate representative sample. The birthplaces of the participants in Guangzhou, other cities in Guangdong province, and other provinces each accounted for approximately one-third of the participants, which might extend the generalizability of the current study. Third, only Cronbach’s alpha was used to assess the reliability of the scale items. More forms of reliability and validity assessment, such as test-retest reliability, and discriminate validity should be conducted in future studies. Finally, the validity of self-reported data should be taken into consideration in any study of sexual behavior. However, an anonymous electronic questionnaire was administered to remove such barriers to participation in this survey [23, 62].

## Conclusion

Our results stressed the need to pay more attention to the relatively lower proportion of consistent condom use among MSM in the current study. Thus, targeted intervention for safe sex should be created. The modified IMB model fitted the data ideally and could serve as a theoretical framework of behavioral interventions for safe sex. According to the final modified IMB model developed in this study, interventions to increase condom use among MSM should prioritize reducing depression level and increasing HIV-related information, motivation, behavioral skills, and access to HIV prevention services by targeting those with lower education levels and incomes, those who have experienced CSA and IPV, and those who seek sexual partners offline. As components of the modified IMB model, the changeable factors can be targeted to improve the effectiveness of the behavioral intervention programs. Nevertheless, further prospective studies are needed to examine the predictive power of the modified IMB model. Furthermore, behavioral intervention programs with a multilevel approach under guideline of the modified IMB model are encouraged to improve consistent condom use among MSM.

## Additional file

Additional file 1: Questionnaire for Modified IMB Model. (DOC 187 kb)

## Abbreviations

AGFI: adjusted goodness of fit index
AIDS: acquired immune deficiency syndrome
ART: antiretroviral therapy
CDC: Centers for Disease Control and Prevention
CSA: child sexual abuse
GFI: goodness of fit index
HIV: human immunodeficiency virus
IMB: Information-Motivation-Behavioral skills
IPV: intimate partner violence
M: mean
MSM: men who have sex with men
RMSEA: root mean square error of approximation
SD: standard deviation
SDS: self-rating depression scale
STI: sexually transmitted infection
UAI: unprotected anal intercourse
VCT: voluntary counseling testing
WLS: weighted least squares
