# Supplementary material for: Predictors of condom use behavior among men who have sex with men in China using a modified information-motivation-behavioral skills (IMB) model
Source: BMC Public Health. 2019 Mar 4;19:261. doi: 10.1186/s12889-019-6593-8 (PMC6399930; doi:10.1186/s12889-019-6593-8)
Supplement: Supplementary file 1 — Questionnaire for Modified IMB Model. (DOC 187 kb) [file 12889_2019_6593_MOESM1_ESM.doc]

| | **Questionnaire for the Modified IMB Model** | | --- | | **1.** A1. Do you have anal/oral intercourses with a man within the past 12 months? | | ○ Yes   ○ No | |  | | | **2.** A2. **Birthday:** ________Year_______Month（e.g.1993Year 02 Month） | | --- | | |  | | **3.** A3. How long have you lived in Guangzhou (months) ?: | | | ○ <3 | ○ 3~6 | ○ 7~12 | ○ >12 |  | | --- | --- | --- | --- | --- | | |  | | **4.** A4. **Birthplace**: | | | ○ Guangzhou City | ○Other cities in Guangdong province |  | ○ Other provinces | | --- | --- | --- | --- | | |  | | **5.** A5. **Marital status**: | | | ○ Unmarried | ○ Married | ○ Cohabitation | ○ Divorced/widowed | | --- | --- | --- | --- | | | **6.** A6. **Education**: | | | ○ Illiteracy | ○ Primary school | ○ Middle school | | --- | --- | --- | | ○ High school or equivalent | ○ College or above |  | | |  | | **7.** A7. **Occupation**： | | | ○ Students | ○ Employed | ○ Part-time |  | | --- | --- | --- | --- | | ○ Freelanced | ○ Unemployed | ○ Retired |  | | |  | | **8.** A8. **Monthly income (Yuan)**: | | | ○ 0 | ○ 1-3000 | ○ 3001-5000 | ○ 5001-10000 | ○ >10000 |  | | --- | --- | --- | --- | --- | --- | | |  | | **9.** A9. What is your sexual orientation? | | | ○ Homosexual | ○ Heterosexual | ○ Bisexual | ○ Unsure |  | | --- | --- | --- | --- | --- | | |  | | **10.** A10. What is your main place/way to find a male sex partner? | | | ○ Bars/Dance halls | ○ Teahouses/Clubs | ○ Public baths |  | | --- | --- | --- | --- | | ○ Parks /Public toilets /Grasslands | ○ Internet | ○ Others |  | | |  | | **11. A11. When you have sex with a man, what is your sex role?**   | ○ Receptive only | ○ Receptive mostly | | ○Receptive and insertive in half | | --- | --- | --- | --- | | ○ Insertive mostly | ○ Insertive only |  | |   **12.** B1. Is AIDS an incurable serious infectious disease? | | ○ Yes   ○ No   ○ Unknown | |  | | **13.** B2. Are men who have sex with men (MSM) the most serious victims of AIDS in China? | | ○ Yes   ○ No   ○ Unknown | |  | | **14.** B3. May a person who looks healthy carry HIV? | | ○ Yes   ○ No   ○ Unknown | |  | | **15.** B4. Will infection with other sexually transmitted diseases increase the risk of HIV infection? | | ○ Yes   ○ No   ○ Unknown | |  | | **16.** B5. Can correct condom use reduce the risk of HIV infection and transmission? | | ○ Yes   ○ No   ○ Unknown | |  | | **17.** B6. Will the use of new drugs (methamphetamine, ecstasy, ketamine, etc.) increase the risk of HIV infection? | | ○ Yes   ○ No   ○ Unknown | |  | | **18.** B7. Should HIV consultation and testing be actively sought after high-risk behaviors (needle-sharing drug abuse/unsafe sex, etc.)? | | ○ Yes   ○ No   ○ Unknown | |  | | **19.** B8. Does deliberately spreading AIDS bear legal liability? | | ○ Yes   ○ No   ○ Unknown | | Questions 20 to 24 ask you to describe your feelings about a specific behavior that involves a male sex partner. If you do not currently have a male sex partner, please answer these questions as if you have one. | | **20. Personal attitudes** | | |  | Very bad | Bad | Neither bad nor good | Good | Very Good | | --- | --- | --- | --- | --- | --- | | **C1a.** Discussion with my sexual partner about safe sex before sexual intercourse during the next month would be | ○ | ○ | ○ | ○ | ○ | | **C2a.** Trying to persuade my partners to practice only safe sex during the next month would be | ○ | ○ | ○ | ○ | ○ | | **C3a.** My having condoms handy (eg. buying condoms) before having sex during the next month would be | ○ | ○ | ○ | ○ | ○ | | **C4a.** Always using condoms during having sex in the next month would be | ○ | ○ | ○ | ○ | ○ | | |  | | **21. Subjective norms** | | |  | Very untrue | Untrue | Neither untrue nor true | True | Very true | | --- | --- | --- | --- | --- | --- | | **C1b.** Most people who are important to me think I should discuss safe sex with a sexual partner before sexual intercourse during the next month. | ○ | ○ | ○ | ○ | ○ | | **C2b.** Most people who are important to me think I should try to persuade my partners to practice only safe sex during the next month | ○ | ○ | ○ | ○ | ○ | | **C3b.** Most people who are important to me think I should have condoms handy before having sex during the next month | ○ | ○ | ○ | ○ | ○ | | **C4b.** Most people who are important to me think my partners and I should always use condoms during having sex in the next month | ○ | ○ | ○ | ○ | ○ | | |  | | **22. Behavioral intentions** | | |  | Very unlikely | Unlikely | Neither unlikely nor likely | Likely | Very likely | | --- | --- | --- | --- | --- | --- | | **C1c.** If I have sex during the next month, I intend to discuss safe sex with a sexual partner before sexual intercourse | ○ | ○ | ○ | ○ | ○ | | **C2c.** If I have sex during the next month, I intend to persuade my partners to practice only safe sex | ○ | ○ | ○ | ○ | ○ | | **C3c.** If I have sex during the next month, I intend to always have condoms handy | ○ | ○ | ○ | ○ | ○ | | **C4c.** If I have sex during the next month, I intend to have my partners and I always use condom | ○ | ○ | ○ | ○ | ○ | | |  | | **23.** **Perceived difficulty** | | |  | Very hard | Fairly hard | Neither hard nor easy | Fairly easy | Very easy | | --- | --- | --- | --- | --- | --- | | **D1.** How hard would it be for you to buy condoms | ○ | ○ | ○ | ○ | ○ | | **D2.** How hard would it be for you to be supportive if your sexual partner brought up the topic of using condoms to reduce the risk of getting HIV? | ○ | ○ | ○ | ○ | ○ | | **D3.** How hard would it be for you to make safer sex with a condom sexually exciting for your partner? | ○ | ○ | ○ | ○ | ○ | | | | **D4.** How hard would it be for you to discuss safe sex with your partner in a non-sexual setting, such as while riding in your car? | ○ | ○ | ○ | ○ | ○ | | --- | --- | --- | --- | --- | --- | | **D5.** How hard would it be for you to consistently use condoms with a partner every time you have a one-night stand? | ○ | ○ | ○ | ○ | ○ | | **D6.** How hard would it be for you to use a condom with your partner while under the influence of alcohol or drugs? | ○ | ○ | ○ | ○ | ○ | | |  | | **24.** **Perceived effectiveness** | | |  | Very ineffectively | Somewhat ineffectively | Neither ineffectively nor effectively | Somewhat effectively | Very effectively | | --- | --- | --- | --- | --- | --- | | **D7.** How effectively could you discuss safe sex with a sexual partner before sexual intercourse ? | ○ | ○ | ○ | ○ | ○ | | **D8.** How effectively could you refuse to have unsafe sex (eg. Without condom)? | ○ | ○ | ○ | ○ | ○ | | **D9.** How effectively could you tell your partner that you want to practice only safe sex if you were about to have sex? | ○ | ○ | ○ | ○ | ○ | | **D10.** How effectively could you convince your partner to practice only safe sex? | ○ | ○ | ○ | ○ | ○ | | **D11.** How effectively could you plan ahead to be sure you always have condoms on hand whenever you have sex? | ○ | ○ | ○ | ○ | ○ | | |  | | **25.** **E1.**Did your intimate partner (spouse, boyfriend or other partners who have had sex with you) ever done these to you ?（Multiple choice question） | | □ threatened to stop helping you with money or housing    □ damaged or destroyed your property    □ threatened to tell others about your sexual orientation    □ verbally threatened to physically harm someone you cared for    □ verbally threatened to harm you physically or emotionally    □ hit you or threw something at you    □ forced you to have unwanted sex    □ Others _________________     □ None | |  | | **26. F1.** Did you have any sexual experiences (someone exposed his genitals or masturbated in front of you or attempted to have or had oral or anal sex with you) before 12 years of age? | | | ○ Yes | ○ No  (Jump to answer Question 28. F3) |  | | --- | --- | --- | | |  | | **27. F2.** Was the person you had the sexual experience with an adult or someone at least 5 years older than you? | | | ○ Yes | ○ No |  | | --- | --- | --- | | |  | | **28. F3.** Between 12 and 16 years of age, did you have any unwanted sexual experiences? | | | ○ Yes | ○ No |  | | --- | --- | --- | | |  | | **29.** **F4.** Between 12 and 16 years of age, did you have any sexual experiences (wanted or unwanted) with an adult or someone who was at least 5 years older than you? | | | ○ Yes | ○ No |  | | --- | --- | --- | | |  | | **30.** **G1.** Do you have anal intercourses with men within the past 6 months ? | | | ○ Yes | ○ No  (Jump to answer Question 32. H1) |  | | --- | --- | --- | | |  | | **31. G2.** How often do you use condom during anal intercourses with men in the last 6 months ? | | ○ Never     ○ Occasionally   ○ Sometimes   ○ Often   ○ Always | |  | | **32.** **H1.** Had you received any HIV prevention services within the last year ? | | |  | Yes | No | | --- | --- | --- | | **H1a** Condom promotion and distribution or HIV voluntary counseling and testing | ○ | ○ | | **H1b** Community-based methadone maintenance treatment or cleaning needle provided or exchanged | ○ | ○ | | **H1c** Peer education for HIV prevention | ○ | ○ | | |  | | **33.** **I.** For each item below, please make a choice which best describes how often you felt or behaved this way during the past seven days. | | |  | A little of the time | Some of the time | Good part of the time | Most of the time | | --- | --- | --- | --- | --- | | **I1.** I feel down-hearted and blue. | ○ | ○ | ○ | ○ | | **I2.** Morning is when I feel the best. | ○ | ○ | ○ | ○ | | **I3.** I have crying spells or feel like it. | ○ | ○ | ○ | ○ | | **I4.** I have trouble sleeping at night. | ○ | ○ | ○ | ○ | | **I5.** . I eat as much as I used to. | ○ | ○ | ○ | ○ | | **I6.** I still enjoy sex. | ○ | ○ | ○ | ○ | | **I7.** I notice that I am losing weight. | ○ | ○ | ○ | ○ | | **I8.** I have trouble with constipation. | ○ | ○ | ○ | ○ | | **I9.** My heart beats faster than usual. | ○ | ○ | ○ | ○ | | **I10.** I get tired for no reason. | ○ | ○ | ○ | ○ | | **I11.** My mind is as clear as it used to be. | ○ | ○ | ○ | ○ | | **I12.** I find it easy to do the things I used to. | ○ | ○ | ○ | ○ | | **I13.** I am restless and can’t keep still. | ○ | ○ | ○ | ○ | | **I14.** I feel hopeful about the future. | ○ | ○ | ○ | ○ | | **I15.** . I am more irritable than usual. | ○ | ○ | ○ | ○ | | **I16.** I find it easy to make decisions. | ○ | ○ | ○ | ○ | | **I17.** . I feel that I am useful and needed. | ○ | ○ | ○ | ○ | | **I18.** My life is pretty full. | ○ | ○ | ○ | ○ | | **I19.** I feel that others would be better off if I were dead. | ○ | ○ | ○ | ○ | | **I20.** I still enjoy the things I used to do. | ○ | ○ | ○ | ○ | | |
| --- | --- | --- | --- | --- | --- | --- | --- | --- | --- | --- | --- | --- | --- | --- | --- | --- | --- | --- | --- | --- | --- | --- | --- | --- | --- | --- | --- | --- | --- | --- | --- | --- | --- | --- | --- | --- | --- | --- | --- | --- | --- | --- | --- | --- | --- | --- | --- | --- | --- | --- | --- | --- | --- | --- | --- | --- | --- | --- | --- | --- | --- | --- | --- | --- | --- | --- | --- | --- | --- | --- | --- | --- | --- | --- | --- | --- | --- | --- | --- | --- | --- | --- | --- | --- | --- | --- | --- | --- | --- | --- | --- | --- | --- | --- | --- | --- | --- | --- | --- | --- | --- | --- | --- | --- | --- | --- | --- | --- | --- | --- | --- | --- | --- | --- | --- | --- | --- | --- | --- | --- | --- | --- | --- | --- | --- | --- | --- | --- | --- | --- | --- | --- | --- | --- | --- | --- | --- | --- | --- | --- | --- | --- | --- | --- | --- | --- | --- | --- | --- | --- | --- | --- | --- | --- | --- | --- | --- | --- | --- | --- | --- | --- | --- | --- | --- | --- | --- | --- | --- | --- | --- | --- | --- | --- | --- | --- | --- | --- | --- | --- | --- | --- | --- | --- | --- | --- | --- | --- | --- | --- | --- | --- | --- | --- | --- | --- | --- | --- | --- | --- | --- | --- | --- | --- | --- | --- | --- | --- | --- | --- | --- | --- | --- | --- | --- | --- | --- | --- | --- | --- | --- | --- | --- | --- | --- | --- | --- | --- | --- | --- | --- | --- | --- | --- | --- | --- | --- | --- | --- | --- | --- | --- | --- | --- | --- | --- | --- | --- | --- | --- | --- | --- | --- | --- | --- | --- | --- | --- | --- | --- | --- | --- | --- | --- | --- | --- | --- | --- | --- | --- | --- | --- | --- | --- | --- | --- | --- | --- | --- | --- | --- | --- | --- | --- | --- | --- | --- | --- | --- | --- | --- | --- | --- | --- | --- | --- | --- | --- | --- | --- | --- | --- | --- | --- | --- | --- | --- | --- | --- | --- | --- | --- | --- | --- | --- | --- | --- | --- | --- | --- | --- | --- | --- | --- | --- | --- | --- | --- | --- | --- | --- | --- | --- | --- | --- | --- | --- | --- | --- | --- | --- | --- | --- | --- | --- | --- | --- | --- | --- | --- | --- | --- | --- | --- | --- | --- | --- | --- | --- | --- | --- | --- | --- | --- | --- | --- | --- | --- | --- | --- | --- | --- | --- | --- | --- | --- | --- | --- | --- | --- | --- | --- | --- | --- | --- | --- | --- | --- | --- | --- | --- | --- | --- | --- | --- | --- | --- | --- | --- | --- | --- | --- | --- | --- | --- | --- | --- | --- | --- | --- | --- | --- | --- | --- | --- | --- | --- | --- | --- | --- | --- | --- | --- | --- | --- | --- | --- | --- | --- | --- | --- | --- | --- | --- | --- | --- | --- | --- | --- | --- | --- | --- | --- | --- | --- | --- | --- | --- | --- | --- |

Question 1 to 3: Inclusion criteria;

Question 4 to 11: Demographics and sexual characteristics;

Question 12 to 19: Information;

Question 20 to 22: Motivation (Question 20 for personal attitudes, Question 21 for subjective norms, Question 22 for behavioral intentions);

Question 23 to 24: Behavioral skills (Question 23 for perceived difficulty, Question 24 for perceived effectiveness);

Question 25: Intimate partner violence;

Question 26 to 29: Child sexual abuse;

Question 30 to 31: Condom use;

Question 32: HIV prevention services;

Question 33: Depression.

All questions were single choice questions except for Question 25.
